# Analysis of Camera Arrays Applicable to the Internet of Things

**DOI:** 10.3390/s16030421

**Published:** 2016-03-22

**Authors:** Jiachen Yang, Ru Xu, Zhihan Lv, Houbing Song

**Affiliations:** 1 School of Electronic Information Engineering, Tianjin University, 92 Weijin Road, Tianjin 300072, China; yangjiachen@tju.edu.cn (J.Y.); xu ru@tju.edu.cn (R.X.); 2 Tianjin International Engineering Institute, Tianjin University, 92 Weijin Road, Tianjin 300072, China; 3 SIAT, Chinese Academy of Science, Shenzhen 518000, China; lvzhihan@gmail.com; 4 Department of Electrical and Computer Engineering, West Virginia University, Montgomery, WV 25136, USA

**Keywords:** Internet of Things, sensors, stereo capture, camera array model, parallel cameras, converged cameras, 3D display

## Abstract

The Internet of Things is built based on various sensors and networks. Sensors for stereo capture are essential for acquiring information and have been applied in different fields. In this paper, we focus on the camera modeling and analysis, which is very important for stereo display and helps with viewing. We model two kinds of cameras, a parallel and a converged one, and analyze the difference between them in vertical and horizontal parallax. Even though different kinds of camera arrays are used in various applications and analyzed in the research work, there are few discussions on the comparison of them. Therefore, we make a detailed analysis about their performance over different shooting distances. From our analysis, we find that the threshold of shooting distance for converged cameras is 7 m. In addition, we design a camera array in our work that can be used as a parallel camera array, as well as a converged camera array and take some images and videos with it to identify the threshold.

## 1. Introduction

The Internet of Things (IoT) is the network of various physical objects embedded with sensors, electronics, software and network connectivity. Various sensors are the base of the realization of IoT, because they are the source of the data. The development of various sensors bolsters IoT [1,2,3]. The data generated by the sensors include sounds, images, temperatures, locations, and so on. Images and videos are one kind of the most common representations of data, which can be used in media and environmental monitoring. Cameras generate images and videos to provide the information, such as the appearances and locations of the targets [4,5], so cameras are needed in IoT.

In terms of camera arrays, there are two categories, including converged arrays and parallel arrays [4,6]. Parallax images can be taken by camera arrays, and they form stereoscopic images with depth sensation, which makes stereo imaging possible [7,8,9]. Then, more information of the things in IoT can be provided than traditional simple cameras. On the other hand, the auto-stereoscopic display is among the important methods of stereoscopic display now [10,11,12,13,14]. It can make images more comfortable. It is meaningful when the images or videos are presented to people. Perfect perceptual quality is necessary because we always try to develop technology to serve people better.

The display of an actual 3D scene by parallel camera arrays is only taken outdoors, which is not sunk concavely [15,16]. Therefore, it is unsatisfactory. However, the display scene taken with converged arrays is produced outside and concavely sunk. This makes the scene look alive; but at the same time, there is a flaw that effects the keystone, and vertical parallax will have a negative impact on viewing quality [15,16].

The comparison of parallel cameras and converged cameras has received much attention, and their pros and cons have been discussed greatly [17]. Most of the relative analyses are based on experiment results, and it is always hard to make the experimental values as precise as the theoretical ones. Although some camera array models are built in theory [18,19,20], the fact that only one direction or one plane is considered and other human factors make the models imperfect [21,22]. Our previous work [23] checked the stereoscopic distortion with varied parameters, including inter-camera distance, shooting distance and camera focal length when acquiring images. In this paper, we focus on the shooting distance and try to find the effect of shooting distance on parallel and converged camera arrays precisely. As the base of the analysis, a parallel camera array model and a simplified converged camera array model are built. Based on these models, we analyze their horizontal and vertical parallax and find the relationship between the parallax and shooting distance threshold of the converged cameras. According to the relationship, we can get the best shooting distance of converged cameras, which is also a threshold to distinguish the differences of parallel and converged cameras. It is helpful to make the choice of which kind of cameras to take the stereo-images. To identify the threshold, we design a camera array that can be used as a parallel camera and a converged camera, then carry out some experiments, using them as the proof of our findings.

In Section 2, we build a parallel array model and a simplified converged array model, then analyze the relation between the convergence distance of the converged array and the parallax. The analysis shows that the converged camera array is appropriate for short-distance shooting. In Section 3, we design a converged camera array, which can be also used as a parallel camera array through angle tuning. Then, we carry out some experiments using the designed camera array to identify the pros and cons of the parallel and converged camera array in Section 4. These experiments also identify that the auto-converged camera array outperforms the parallel one if the shooting distance varies in a short-distance range. In Section 5, we give the conclusion and an outlook for future work. Generally speaking, the main contribution of our paper is that we did a detailed analysis about the two kinds of camera arrays’ performance over different shooting distances. The work can benefit the application of the camera arrays in different environments. Another point is the realization of the camera array, which can be used as a parallel camera array, as well as a converged camera array. This is convenient for the different choices according to the real needs.

## 2. Camera Array Models

### 2.1. Converged Camera Array Model

**Figure 1:**
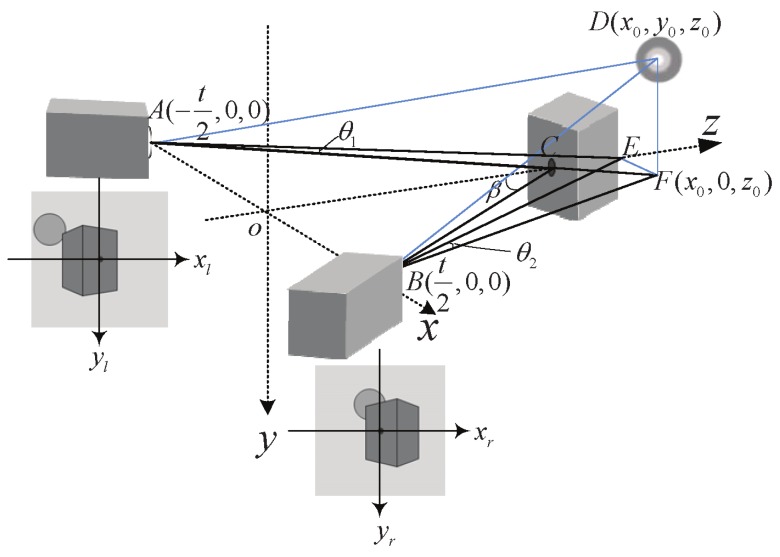
Camera array schematic diagram.

We build a simplified converged model, as Figure 1 shows. *A* and *B* are the optical center of the two cameras. *C* is a reference object, which lies on the *z* axis, and AC, BC are the cameras’ optical axes. *D* is the target object, which we want to display. *F* is the projection of object *D* on the x-z plane. *E* is the point that *F* projects on the *z* axis, so EF//OA. According to the geometry, Equation (1) can be obtained, where *L* indicates convergence distance, *t* indicates the distance between *A* and *B* and *β* represents the converged deflection angle.

(1) $$\begin{array}{c} \tan\beta =\frac{t}{2L} \end{array}$$

There are two kinds of coordinate systems used in Figure 1, the world coordinate system and the image coordinate system. $\left(x_{0},y_{0},z_{0}\right)$ in the world coordinate system is a point in an object plane. Its projections in the right and left camera are correspondingly $\left(x_{r},y_{r}\right)$ and $\left(x_{l},y_{l}\right)$ in the image coordinate system. $\theta_{1}$ is the angle between AE and AF; $\theta_{2}$ is the angle between BE and BF. Then, we can get:

(2) $$\begin{array}{c} \left\{\begin{array}{c} \theta_{1}=\beta -\arctan\left(\frac{t+2x_{0}}{2z_{0}}\right)\\ \theta_{2}=\arctan\left(\frac{t-2x_{0}}{2z_{0}}\right)-\beta \\ \frac{x_{l}}{f}=\tan\theta_{1}\\ \frac{x_{r}}{f}=\tan\theta_{2} \end{array}\right. \end{array}$$

$x_{l}$ and $x_{r}$ can be obtained according to Equation (2),

(3) $$\begin{array}{c} \left\{\begin{array}{c} x_{l}=f\tan\left[\beta -\arctan\left(\frac{t+2x_{0}}{2z_{0}}\right)\right]\\ x_{r}=f\tan\left[\arctan\left(\frac{t-2x_{0}}{2z_{0}}\right)-\beta \right] \end{array}\right. \end{array}$$

AF is obtained after extending AC; then, we can use the geometric similarity to get:

(4) $$\begin{array}{c} \frac{y_{l}}{f}=-\frac{y_{0}}{AF} \end{array}$$

(5) $$\begin{array}{c} AF=z_{0}\cos\beta +\left(x_{0}+\frac{t}{2}\right)\sin\beta \end{array}$$

According to Equations (4) and (5),

(6) $$\begin{array}{c} y_{l}=-\frac{y_{0}f}{z_{0}\cos\beta +\left(x_{0}+\frac{t}{2}\right)\sin\beta } \end{array}$$

(7) $$\begin{array}{c} y_{r}=-\frac{y_{0}f}{z_{0}\cos\beta -\left(x_{0}-\frac{t}{2}\right)\sin\beta } \end{array}$$

To match with the display coordinate system, the image system needs to be expanded *M* times. For points in the display screen $\left(x_{sl},y_{sl}\right)$ and $\left(x_{sr},y_{sr}\right)$, we could get:

(8) $$\begin{array}{c} x_{sl}=Mx_{l},y_{sl}=My_{l},x_{sr}=Mx_{r},y_{sr}=My_{r} \end{array}$$

Then, we get the horizontal and vertical parallax, *h* and *v*, respectively, with $\left(x_{sr},y_{sr}\right)$ and $\left(x_{sl},y_{sl}\right)$,

(9) $$\begin{array}{c} h=x_{sr}-x_{sl},v=y_{sr}-y_{sl} \end{array}$$

**Figure 2:**
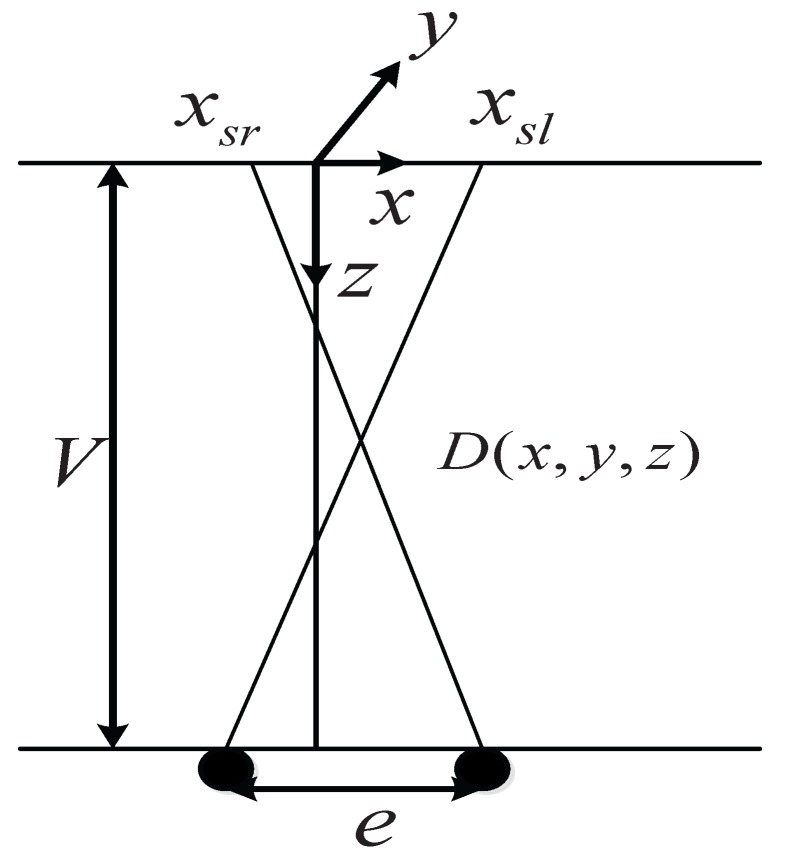
Stereopsis diagram.

For a point (x,y,z) on a stereo image in the image coordinate system and a view distance *V* (seen in Figure 2), we can obtain the following relationship based on the human eye imaging characteristics.

(10) $$\begin{array}{c} \left\{\begin{array}{c} \;\frac{h}{e}=\frac{z}{V-z}\\ \;\frac{V}{V-z}=\frac{x_{sl}+x_{sr}}{2x}\\ \frac{V}{V-z}=\frac{y_{sl}+y_{sr}}{2y} \end{array}\right. \end{array}$$

According to Equations (3)–(10), we can get *h*, *v*, *x*, *y*, *z* as follows.

(11) $$\begin{array}{c} h=Mf\{\tan\left[\arctan\left(\frac{t-2x_{0}}{2z_{0}}\right)-\beta \right]-\tan\left[\beta -\arctan\left(\frac{t+2x_{0}}{2z_{0}}\right)\right]\} \end{array}$$

(12) $$\begin{array}{c} v=\frac{My_{0}f}{z_{0}\cos\beta +\left(x_{0}+\frac{t}{2}\right)\sin\beta }-\frac{My_{0}f}{z_{0}\cos\beta -\left(x_{0}-\frac{t}{2}\right)\sin\beta } \end{array}$$

(13) $$\begin{array}{c} x=\frac{eMf\{\tan\left[\beta -\arctan\left(\frac{t+2x_{0}}{2z_{0}}\right)\right]+\tan\left[\arctan\left(\frac{t-2x_{0}}{2z_{0}}\right)-\beta \right]\}}{2(e+h)} \end{array}$$

(14) $$\begin{array}{c} y=-\frac{eMfy_{0}}{2(e+h)}\left[\frac{1}{z_{0}\cos\beta +\left(x_{0}+\frac{t}{2}\right)\sin\beta }+\frac{1}{z_{0}\cos\beta -\left(x_{0}-\frac{t}{2}\right)\sin\beta }\right] \end{array}$$

(15) $$\begin{array}{c} z=\frac{VMf}{e+h}\left\{\tan\left[\arctan\left(\frac{t-2x_{0}}{2z_{0}}\right)-\beta \right]-\tan\left[\beta -\arctan\left(\frac{t+2x_{0}}{2z_{0}}\right)\right]\right\} \end{array}$$

### 2.2. Parallel Camera Array Model

In the converged model, if the convergence distance *L* approaches infinity, the converged deflection angle *β* will approach zero. That means the camera arrays can be turned into parallel cameras. Then, the relationship in the parallel model can be easily obtained based on what we have in the converged model.

(16) $$\begin{array}{c} \left\{\begin{array}{c} h=\frac{Mft}{z_{0}}\\ v=0\\ \;x=-\frac{eMfx_{0}}{ez_{0}+Mft}\\ \;y=-\frac{eMfy_{0}}{ez_{0}+Mft}\\ z=-\frac{VMft}{ez_{0}+Mft} \end{array}\right. \end{array}$$

### 2.3. Model Analysis

In stereoscopic observation, vertical parallax is unfavorable. In Equation (16), we can see that in images taken by parallel camera arrays, the vertical parallax is zero theoretically, even though physical camera errors and many other kinds of external factors may change the ideal value; while for converged arrays, the vertical parallax is:

(17) $$\begin{array}{c} v=\frac{2tfM\sqrt{4L^{2}+t^{2}}x_{0}y_{0}}{{\left(2Lz_{0}+\frac{t^{2}}{2}\right)}^{2}-{\left(tx_{0}\right)}^{2}} \end{array}$$

As we consider that t<<L,

(18) $$\begin{array}{c} v=\frac{tfMx_{0}y_{0}}{L{z_{0}}^{2}} \end{array}$$

For the converged camera array model, the vertical parallax *v* diminishes as convergence distance *L* increases. As a matter of fact, the viewing angle of the camera lens usually meets $x_{0}<z_{0}$, $y_{0}<z_{0}$, and *L* is usually greater than 1 m. If we set *t* as 70 mm, *f* as 6.5 mm, *M* as 50 and assume the range of *L* is from 1 m to 10 m, *v* will be:

(19) $$\begin{array}{c} 0.00228m\leq v=\frac{tfm}{L}\leq 0.02275m \end{array}$$

This means the converged camera arrays can have a vertical parallax of more than 1 cm, which is enough to have a negative effect on stereoscopy in theory.

**Figure 3:**
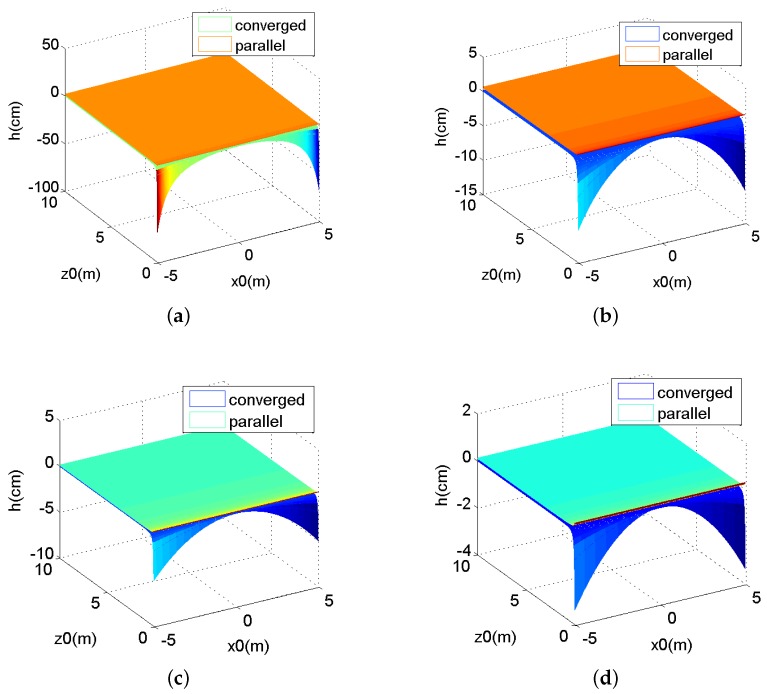
The curve of horizontal parallax *h* and $x_{0}$, $z_{0}$. (**a**) L= 1 m; (**b**) L = 4 m; (**c**) L = 7 m; (**d**) L = 10 m.

In terms of horizontal parallax *h*, it only depends on $z_{0}$ with an inverse relationship for parallel camera arrays, as Equation (16) shows; while for converged camera arrays, *h* is related to $x_{0}$, $z_{0}$ and *L*, as Equation (11) shows. The corresponding relations for parallel and converged camera arrays are illustrated in Figure 3. We set *t* as 70 mm, *f* as 6.5 mm and *M* as 50. *L* is a variable. We choose four values of *L*, 1 m, 4 m, 7 m and 10 m as samples.

From Figure 3, we can see that for the converged array, the horizontal parallax *h* is more and more similar to the *h* of parallel camera arrays in three conditions: when the convergence distance *L* gradually becomes greater, when $|x_{0}|$ becomes smaller or when $z_{0}$ is larger.

**Figure 4:**
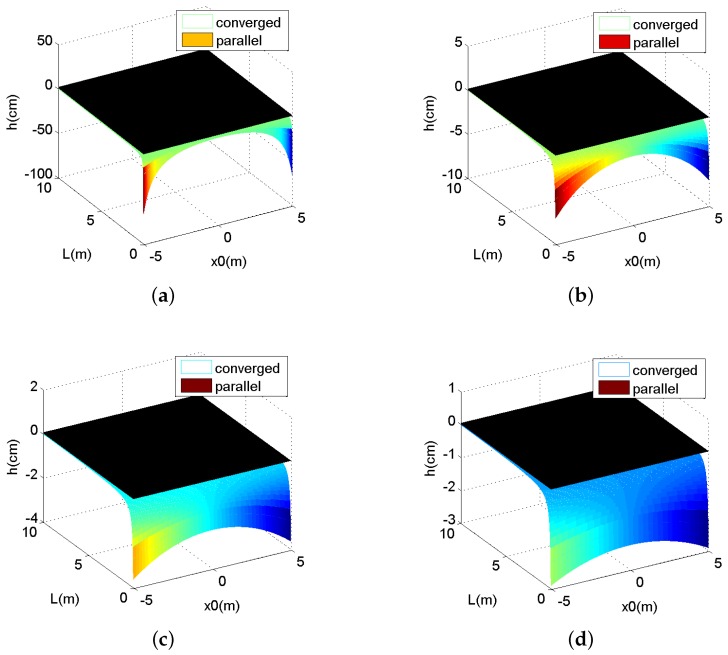
The curve of horizontal parallax *h* and $x_{0}$, *L*. (**a**) $z_{0}=1$ m; (**b**) $z_{0}=4$ m; (**c**) $z_{0}=7$ m; (**d**) $z_{0}\;=\;10$ m.

In another view, the differences of the parallax effect between these two camera arrays can be analyzed, as $z_{0}$ is set as a fixed value. Figure 4 shows corresponding relations, and the four chosen values of $z_{0}$ are 1 m, 4 m, 7 m and 10 m. From these relation curves, we can see in the same condition, as the shooting distance $z_{0}$ becomes longer, the *h* of converged camera arrays is more similar to the *h* of parallel ones.

As shown in Figure 3 and Figure 4, when the convergence distance *L* is small, converged arrays outperform parallel arrays because the negative and positive parallax existing in converged stereo images makes depth sensation more obvious. This is very important in a 3D display. On the other hand, when *L* is large, there is little difference between these two arrays, and both of their depth sensations are unimpressive.

Based on the horizontal parallax threshold, when *L* is longer than 7 m, the horizontal parallax *h* is so small, that the stereo sense dies away. On the other hand, when shooting distance $z_{0}$ is greater than 7 m, converged and parallel camera arrays are almost the same. In conclusion, when the shooting distance is shorter than 7 m, converged arrays are preferable for the scenes taken and 3D display, but when it is greater than 7 m, parallel arrays will stand out as a more suitable option.

## 3. Auto-Converged Camera Array Realization

**Figure 5:**
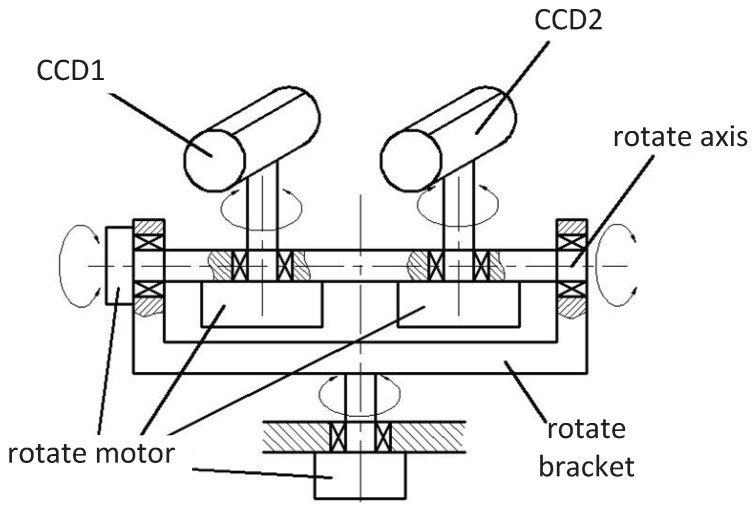
The sensor configuration of the auto-converged camera array.

To identify the analysis in Section 2, we design an auto-converged camera array. The configuration in Figure 5 is made up of two CCD sensors spaced 7 cm apart, pan-tilt-zoom, four stepping motors, rotate axis and rotary bracket. There are four stepping motors that are used to control the four degrees of free rotary movement to simulate the configuration turning left or right and the cameras’ switching angle of view. For accurate fusion of spatial sensor information, proper calibration among the cameras is required. The entire sensor is mounted on a tripod beside the computer. In this paper, we adopt the Kalman filter to track the target [24]. The designed cameras can converge and focus on moving targets automatically based on the measured distance.

**Figure 6:**
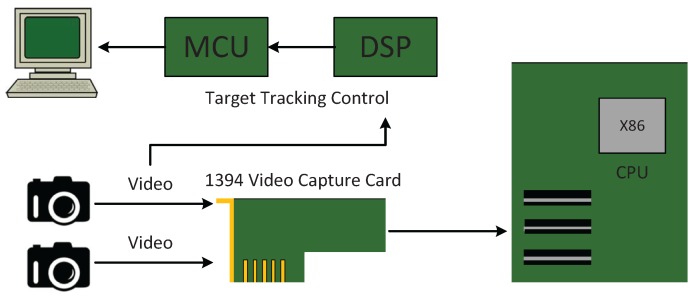
Component interconnections.

An experimental test-bed was configured using multimedia PC components, Microcontroller Unit (MCU) and Digital Signal Processor (DSP). A diagram of the sensor, date acquisition and processing components is shown in Figure 6. The multimedia 1394 video capture card can be used to grasp 1024×768 pixel color images from either camera. Image processing and camera control are performed in real-time on the DSP and MCU.

**Figure 7:**
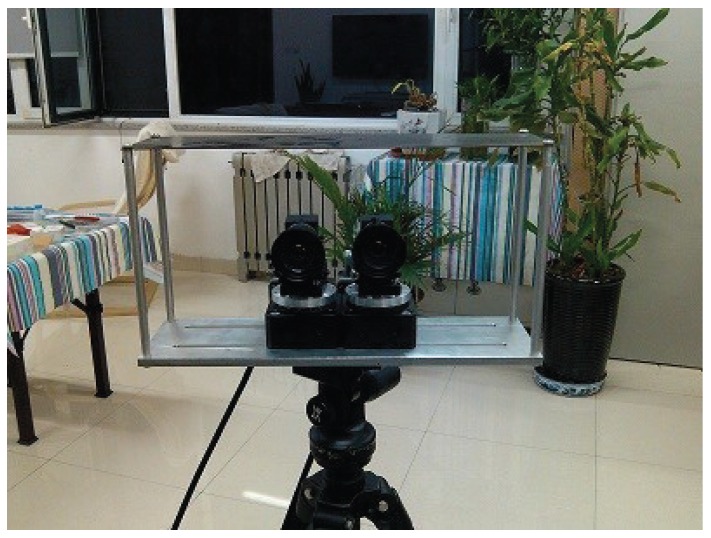
The physical diagram of the auto-converged camera array.

**Figure 8:**
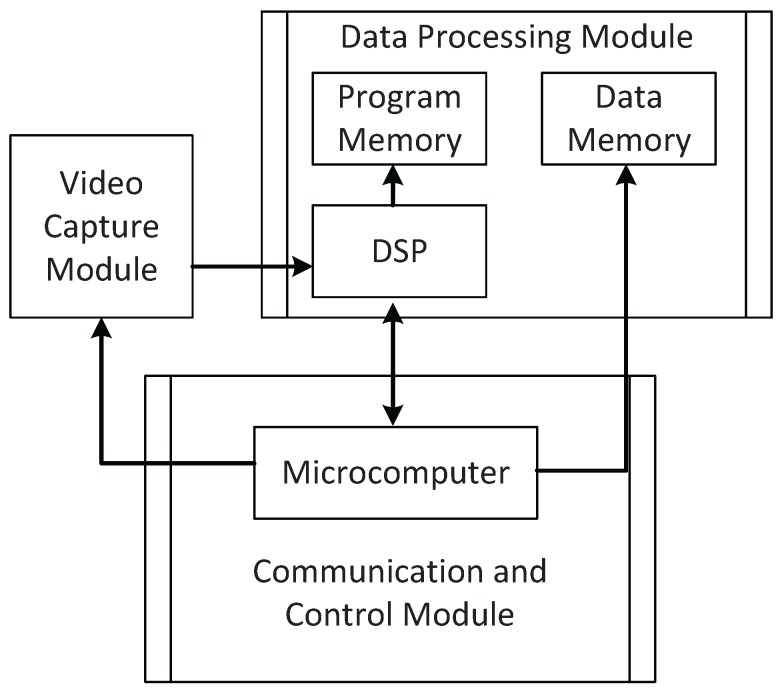
Structure of the system hardware.

The general working principle of the system is: firstly, digital images captured by cameras are processed with the DSP chip; secondly, the result of processing is transmitted to the microcomputer through the bus; thirdly, the movement of the camera system is controlled with a stepper motor driven by the microcomputer. As shown in Figure 7, the system consists of the video capture module, the data processing module and the communication and control module. Figure 8 shows the physical frame of the designed auto-converged camera array.

Auto-converged arrays are widely used in three-dimensional reconstructions, face recognition, gesture recognition and image assessment based on stereo vision, and so on. In image assessment, stereo images are necessary for the experiments. Auto-converged camera arrays can be a perfect tool to take stereo images for the study of image assessment, as Yang *et al.* do in [25].

## 4. Auto-Converged Experiment and Measure

**Figure 9:**
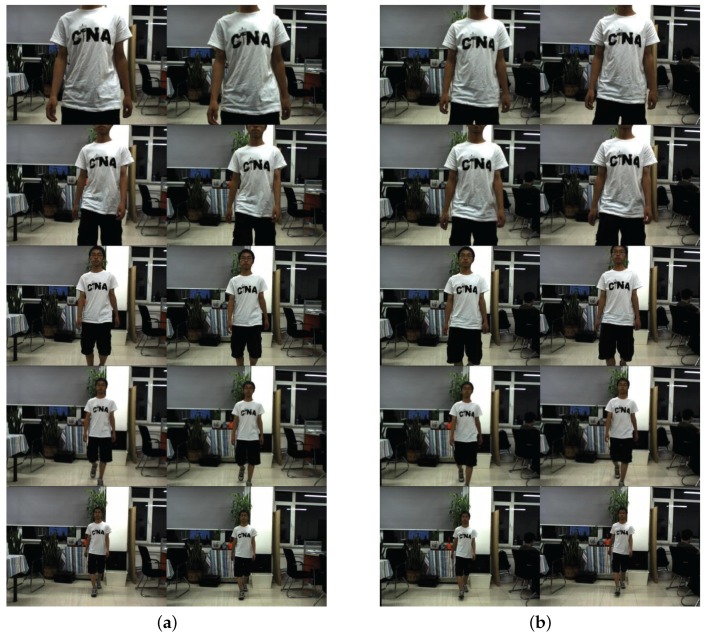
Images taken by using the converged camera array and the parallel camera array. (**a**) Images taken by using the converged camera array; (**b**) images taken by using the parallel camera array.

In order to verify the analysis above, we use a parallel and a converged camera array to shoot images of a moving man as the target in the same condition, then contrast these two groups of images, as shown in Figure 9. The resolution of the camera array used is 1024×768. The convergence distance varies in the range of 1 m to 10 m.

We study the stereo images taken by the parallel array and find that the stereo sense is obvious if the shooting distance is large, but for a short distance, the images cannot be fused. To be more precise, if the shooting distance is less than 3 m, it is difficult for the obtained parallax images to be fused; if it is between 3 m and 4 m, fusing is realized, but there exists much ghosting; if it goes beyond 4 m and up to 10 m, better fusing is realized with very little ghosting, but it is still not enough to realize the perfect life-like effect of the actual three-dimensional scene.

Equation (16) shows that horizontal parallax from parallel images decreases as shooting distance increases. If shooting distance $z_{0}$ is less than 3 m, the fusion of parallax images cannot be realized, as the horizontal parallax goes beyond the allowed range; if $z_{0}$ varies from 3 m to 4 m, the images can be fused, but there exists much ghosting; if $z_{0}$ is greater than 4 m, the parallax is in the allowed range, and the corresponding images fuse perfectly. However, only positive parallax appears in fused images, so only a protruding visual effect can be obtained.

We study converged stereo-images and find that if the shooting distance is less than 5 m, stereo images taken by the converged array have a good stereo sense; if it changes from 5 m to 7 m, the visual stereo perception decreases; if it is greater than 7 m, the converged camera array works almost the same as the parallel camera array. It makes no perceptual difference between them. Therefore, 7 m is the threshold to distinguish the difference of the parallel and converged camera arrays. The three-dimensional scene can be displayed in a life-like manner with the images taken by the converged array, which is protruding outside and concavely sunk.

We use the designed camera array to take two groups of pictures of a man moving slowly toward the camera. One group is taken by a parallel camera array, and the other is taken by a converged one. The shooting distance for both cameras in the experiment varies in the range from 1 m to 10 m. We take integers of the distance as our experiment setting and take five pictures at every distance. Then, we get 50 pictures for the parallel array and 50 pictures for the converged one. Figure 9 gives some examples of the pictures taken. The observers feel dizzy when they look at the parallel stereo images. Their eyes will be tired if the scene taken in the pictures is close to the cameras; while stereoscopic images taken by the parallel camera array offer us a comfortable depth sensation, and the target keeps in near the center of the imaging plane. The converged camera array reproduces the true three-dimensional scene.

We also adopt the objective evaluation method for stereo image quality to evaluate the stereo images based on the algorithm called DIIVINE in [26]. Table 1 gives the corresponding results. We can see from the figures in the table that when the shooting distance is less than 7 m, the overall performance of the converged cameras is better than the parallel cameras, while when the shooting distance is more than 7 m, they are comparable. Combined with the analysis above, we can conclude that converged camera arrays outperform parallel camera arrays when the shooting distance is less than 7 m; however, if we take the keystone distortion into consideration, parallel camera arrays will be better in the condition of a shooting distance greater than 7 m.

## 5. Conclusions

In this paper, we build a parallel camera array model and a simplified converged camera array model as the base of subsequent analysis. A physical camera array is developed, which can be used as a parallel array, as well as a converged array. This is convenient for the different choices according to the real needs. The pros and cons of these two kinds of cameras are discussed. A conclusion is that converged arrays are more suitable for short-distance shooting and parallel arrays for long-distance shooting. Our work can be guidance to the application of the camera arrays in different environments. For future work on the auto-converged camera array, we need to focus on camera calibration and visual stereo-video evaluation.

## Figures and Tables

**Table 1:** DIIVINE quality of the images taken by the converged and the parallel camera array.

Shooting Distance	1 m	2 m	3 m	4 m	5 m
converged	0.75	0.79	0.82	0.84	0.86
parallel	0.60	0.67	0.75	0.79	0.81
shootingdistance	6 m	7 m	8 m	9 m	10 m
converged	0.86	0.83	0.81	0.82	0.80
parallel	0.83	0.83	0.82	0.83	0.81
